# Association of race/ethnicity with mortality in patients hospitalized with COVID-19

**DOI:** 10.1371/journal.pone.0267505

**Published:** 2022-08-04

**Authors:** Safiya Richardson, Johanna Martinez, Jamie S. Hirsch, Jane Cerise, Martin Lesser, Robert O. Roswell, Karina W. Davidson, the Northwell Health COVID-19 Research Consortium

**Affiliations:** 1 Institute of Health Innovations and Outcomes Research, Feinstein Institutes for Medical Research, Northwell Health, Manhasset, NY, United States of America; 2 Donald and Barbara Zucker School of Medicine at Hofstra/Northwell, Northwell Health, Hempstead, NY, United States of America; 3 Department of Information Services, Northwell Health, New Hyde Park, NY, United States of America; 4 Biostatistics Unit, Feinstein Institutes for Medical Research, Northwell Health, Manhasset, NY, United States of America; 5 Institute of Health System Science, Feinstein Institutes for Medical Research, Northwell Health, New Hyde Park, NY, United States of America; Stony Brook University Renaissance School of Medicine, UNITED STATES

## Abstract

**Objective:**

To evaluate racial and ethnic differences in mortality among patients hospitalized with coronavirus disease 2019 (COVID-19) after adjusting for baseline characteristics and comorbidities.

**Methods:**

This retrospective cohort study at 13 acute care facilities in the New York City metropolitan area included sequentially hospitalized patients between March 1, 2020, and April 27, 2020. Last day of follow up was July 31, 2020. Patient demographic information, including race/ethnicity and comorbidities, were collected. The primary outcome was in-hospital mortality.

**Results:**

A total of 10 869 patients were included in the study (median age, 65 years [interquartile range (IQR) 54–77; range, 18–107 years]; 40.5% female). In adjusted time-to-event analysis, increased age, male sex, insurance type (Medicare and Self-Pay), unknown smoking status, and a higher score on the Charlson Comorbidity Index were significantly associated with higher in-hospital mortality. Adjusted risk of hospital mortality for Black, Asian, Hispanic, multiracial/other, and unknown race/ethnicity patients were similar to risk for White patients.

**Conclusions:**

In a large diverse cohort of patients hospitalized with COVID-19, patients from racial/ethnic minorities experienced similar mortality risk as White patients.

## Introduction

Age-adjusted, nationwide, publicly reported data has demonstrated increased rates of coronavirus disease 2019 (COVID-19) mortality for all racial and ethnic minorities in the United States [1]. Compared to non-Hispanic Whites, non-Hispanic Blacks are 3.6 times more likely to die from this disease, Hispanics 2.8 times more likely, non-Hispanic American Indian/Alaska Natives 2.2 times more likely, and non-Hispanic Asian or Pacific Islanders are 1.6 times more likely. These striking disparities have received national attention as several states, cities, and healthcare systems have struggled to gather accurate data and identify potential interventions [2–4].

Said disparities are the consequence of structural racism—the totality of ways in which societies foster racial discrimination through mutually reinforcing systems of housing, education, employment, earnings, benefits, credit, media, health care, and criminal justice [5]. Potential modifiers include differential access to primary care, exposure to the public in essential work, English language proficiency, health worker bias, and rates of chronic illness. Based on risk factors identified by the Centers for Disease Control (including chronic lung disease, diabetes and heart disease), racial and ethnic minorities are at higher risk for severe COVID-19 illness [6].

Published analyses of disparities in in-hospital mortality have almost entirely focused on non-Hispanic Blacks and found that, although they are more likely to acquire and be hospitalized for COVID-19, they are not more likely to experience in-hospital mortality [7–12]. One study that evaluated in-hospital mortality rates in a population of predominantly Hispanics and non-Hispanic Blacks found no difference compared to White patients [13]. Another study evaluated in-hospital mortality rates in a diverse population and found that Blacks were less likely to experience in-hospital mortality compared to Whites [14].

Published work examining racial/ethnic disparities in in-hospital mortality controlled for appropriate covariates is limited. Data evaluating disparities in diverse populations including considerable numbers of patients with Hispanic and non-Hispanic Asian race/ethnicities is particularly limited. This study evaluates mortality rates in diverse patients hospitalized with COVID-19 in the New York City area by race/ethnicity, adjusted for baseline characteristics and comorbidities.

## Materials and methods

This retrospective cohort study was conducted at a large academic health system in New York, serving approximately 11 million persons in Long Island, Westchester County, and New York City. The population served is 49% non-Hispanic White, 23% Hispanic, 13% non-Hispanic Asian, 12% non-Hispanic Black, and 3% non-Hispanic multiracial and other. The Northwell Health Institutional Review Board approved this study as minimal-risk research using data collected for routine clinical practice and waived the requirement for informed consent. This study followed the Strengthening the Reporting of Observational Studies in Epidemiology (STROBE) reporting guidelines [15].

The study included patients 18 years and older admitted to and discharged from one of 13 acute care facilities with confirmed COVID-19 between March 1, 2020, and April 27, 2020, inclusive of those dates. COVID-19 was confirmed by nasopharyngeal polymerase chain reaction testing. Clinical outcomes were monitored until July 31, 2020, the final date of follow-up. Pregnant patients admitted during the study period for labor and delivery were excluded from the study as these patients were asymptomatically screened for COVID-19. Of the 11 159 adult patients admitted during the study period, 290 were excluded as labor and delivery patients who were asymptomatically screened for COVID-19. The final study cohort included the remaining 10 869 patients. Although several studies using variations of this cohort of patients have been published, none examined disparities in in-hospital mortality by patient race/ethnicity [16–20].

### Data collection

Data were collected from the enterprise electronic health record (EHR; Sunrise Clinical Manager, Allscripts, Chicago, IL, United States [U.S.]) reporting database. Data collected included patient demographic information (age, sex, race, ethnicity, insurance type), comorbidities (as captured in the database by International Classification of Disease coding for chronic medical conditions), and outcomes including discharge, length of stay, and death. Each patient’s Charlson Comorbidity Index (CCI) was calculated using data collected from baseline comorbidities. The CCI predicts 10-year survival in patients with multiple comorbidities and was used as a measure of total comorbidity burden [21].

Race and ethnicity data were collected by self-report in pre-specified, fixed categories. Patients who reported themselves as Hispanic in ethnicity were categorized as Hispanic, regardless of reported race. The remaining non-Hispanic patients were categorized based on race. Patients without reported ethnicity were also categorized based on race alone. Those without race or ethnicity data were categorized as unknown. Patients with self-reported race as Native Hawaiian, Pacific Islander, Native American, Alaska Native, or multiracial were classified as multiracial/other. This categorization process resulted in 6 groups: non-Hispanic White, Hispanic, non-Hispanic Black, non-Hispanic multiracial or other, non-Hispanic Asian, and unknown.

Transfers from one in-system hospital to another were merged and considered as one visit. Transfers out of the system to another acute care facility were considered censored at outside transfer date. Transfers out of the system to another acute care facility were conducted to offload high-census hospitals during the peak of the COVID-19 pandemic. For patients with any readmission during the study period, only the first visit was considered in the analysis. Patients still admitted to the hospital at the last day of follow-up were considered censored at that date.

### Statistical analysis

Descriptive statistics were reported for each variable stratified by race/ethnicity. Continuous variables were reported as means and standard deviations, medians and interquartile ranges, and ranges. Categorical variables are reported as frequencies and percentages. Analysis of variance was used to test for association between race/ethnicity and continuous variables. Chi-square tests were used to test for association between categorical variables and race/ethnicity followed by paired comparisons between the reference group and other racial/ethnic groups.

A Cox proportional hazard (PH) model was used to examine the relationship between race/ethnicity and in-hospital mortality both with and without adjusting for several demographic and clinical factors. Covariates were selected a priori and on the basis of clinical relevance. The candidate variables for inclusion in the model were age group, gender, race/ethnicity, insurance type, hypertension, asthma, CCI group, and smoking status as well as the interactions between race/ethnicity and age group and race/ethnicity and gender (as said interactions were deemed of particular interest). Asthma and hypertension were included in the model in addition to CCI score, as these medical conditions are not included in the CCI and have been of particular interest in COVID-19. For any factors that violated the proportional hazards assumption, a factor x time interaction term was introduced into the model. Backward elimination was used to select the final adjusted model. A p-value < 0.05 was considered statistically significant. All analyses were conducted using Statistical Analysis System (SAS) version 9.4 (SAS Institute Inc., Cary, NC).

## Results

Of the 11 159 adult patients admitted during the study period, 290 were excluded as labor and delivery patients who were asymptomatically screened for COVID-19. The final study cohort included the remaining 10 869 patients (median age, 65 years [IQR 54–77; range, 18–107 years]; 40.5% female) (**Table 1**). At the study end point, 7747 (71.3%) of patients included in the study had been discharged alive, 2725 (25.1%) had expired, 6 (0.0006%) were still in hospital, and 391 (3.6%) had been transferred to an outside acute care facility. The study sample included 3700 (34%) White patients, 2310 (21.3%) Hispanic patients, 2293 (21.1%) Black patients, 1206 (11.1%) Multi/Other patients, 935 (8.6%) Asian patients, and 425 (3.9%) patients with unknown race or ethnicity.

**Table 1:** Baseline characteristics of patients hospitalized with COVID-19.

	Total	Non-Hispanic White^a^	Hispanic or Latino	Non-Hispanic Black	Non-Hispanic Multi/Other	Non-Hispanic Asian	Unknown	p-value**
*Demographic Information*, *N or n(%)*	N = 10,869	3700 (34.0%)	2310 (21.3%)	2293 (21.1%)	1206 (11.1%)	935 (8.6%)	425 (3.9%)	
**Age, yr, median (IQR)**	65.0 (54–77)	72.0 (61–82)	59.0 (48–71)	65.0 (55–75)	62.0 (52–72)	63.0 (52–73)	63.0 (51–75)	< 0.001
**Age Group, yr**								
18–39	779 (7.2)	153 (4.1)	261 (11.3)	170 (7.4)	105 (8.7)	63 (6.7)	27 (6.4)	
40–49	1096 (10.1)	197 (5.3)	357 (15.5)	209 (9.1)	155 (12.9)	119 (12.7)	59 (13.9)	
50–59	2038 (18.8)	476 (12.9)	548 (23.7)	453 (19.8)	264 (21.9)	205 (21.9)	92 (21.7)	
60–69	2522 (23.2)	758 (20.5)	514 (22.3)	603 (26.3)	308 (25.5)	240 (25.7)	99 (23.3)	< 0.001
70–79	2268 (20.9)	931 (25.2)	373 (16.2)	491 (21.4)	213 (17.7)	179 (19.1)	81 (19.1)	
80 and up	2166 (19.9)	1185 (32.0)	257 (11.1)	367 (16.0)	161 (13.4)	129 (13.8)	67 (15.8)	
**Gender**								
Female	4397 (40.5)	1535 (41.5)	830 (35.9)	1097 (47.8)	452 (37.5)	334 (35.7)	149 (35.1)	< 0.001
Male	6472 (59.6)	2165 (58.5)	1480 (64.1)	1196 (52.2)	754 (62.5)	601 (64.3)	276 (64.9)	
**Insurance**								
Commercial	3233 (29.8)	981 (26.5)	610 (26.4)	775 (33.8)	415 (34.4)	343 (36.7)	109 (25.7)	< 0.001
Medicaid	2212 (20.4)	262 (7.1)	891 (38.6)	375 (16.4)	324 (26.9)	235 (25.1)	125 (29.4)	
Medicare	5122 (47.1)	2419 (65.4)	698 (30.2)	1084 (47.3)	423 (35.12)	343 (36.7)	155 (36.5)	
Self-pay	180 (1.7)	15 (0.4)	79 (3.4)	28 (1.2)	24 (2.0)	6 (0.6)	28 (6.6)	
Other^b^	122 (1.1)	23 (0.6)	32 (1.4)	31 (1.4)	20 (1.7)	8 (0.9)	8 (1.9)	
***Comorbidities*, *n(%)***								
**Cancer**	831 (7.7)	419 (11.3)	95 (4.1)	184 (8.0)	55 (4.6)	59 (6.3)	19 (4.5)	< 0.001
**Cardiovascular Disease**	6789 (62.5)	2429 (65.7)	1154 (50.0)	1671 (72.9)	696 (57.7)	598 (64.0)	241 (56.7)	< 0.001
Hypertension	6453 (59.4)	2233 (60.4)	1110 (48.1)	1623 (70.8)	674 (55.9)	581 (62.1)	232 (54.6)	< 0.001
Coronary Artery Disease	1393 (12.8)	680 (18.4)	174 (7.5)	213 (9.3)	149 (12.4)	138 (14.8)	39 (9.2)	< 0.001
Congestive Heart Failure	876 (8.1)	405 (11.0)	110 (4.8)	216 (9.4)	63 (5.2)	57 (6.1)	25 (5.9)	< 0.001
Peripheral Vascular Disease	274 (2.5)	129 (3.5)	44 (1.9)	58 (2.5)	27 (2.2)	8 (0.9)	8 (1.9)	< 0.001
**Chronic Liver Disease** ^**c**^	283 (2.6)	90 (2.4)	59 (2.6)	65 (2.8)	33 (2.7)	30 (3.2)	6 (1.4)	0.45
**Chronic Respiratory Disease**	1447 (13.3)	600 (16.2)	239 (10.4)	327 (14.3)	130 (10.8)	103 (11.0)	48 (11.3)	< 0.001
Asthma	892 (8.2)	274 (7.4)	192 (8.3)	229 (10.0)	93 (7.7)	74 (7.9)	30 (7.1)	0.015
Chronic Obstructive Pulmonary Disease	649 (6.0)	365 (9.9)	63 (2.7)	122 (5.3)	43 (3.6)	37 (4.0)	19 (4.5)	< 0.001
**Diabetes Mellitus** ^**d**^	3923 (36.1)	1087 (29.4)	806 (34.9)	1022 (44.6)	456 (37.8)	415 (44.4)	137 (32.2)	< 0.001
**Kidney Disease**	887 (8.2)	306 (8.3)	128 (5.5)	279 (12.2)	80 (6.6)	67 (7.2)	27 (6.4)	< 0.001
Chronic Kidney Disease^e^	421 (3.9)	205 (5.5)	39 (1.7)	101 (4.4)	34 (2.8)	28 (3.0)	14 (3.3)	< 0.001
End-Stage Kidney Disease^f^	466 (4.3)	101 (2.7)	89 (3.9)	178 (7.8)	46 (3.8)	39 (4.2)	13 (3.1)	< 0.001
**Charlson comorbidity index** ^**g**^ **, median (IQR)**	4 (2–7)	6 (3–8)	3 (1–5)	5 (3–7)	3 (2–6)	4 (2–6)	3 (2–6)	< 0.001
**Charlson comorbidity index**^**g**^ **group**								
0	921 (8.5)	177 (4.8)	345 (14.9)	133 (5.8)	136 (11.3)	85 (9.1)	45 (11.0)	< 0.001
1–2	2250 (20.7)	485 (13.1)	699 (30.3)	400 (17.4)	331 (27.5)	224 (24.0)	111 (26.1)	
3–4	2558 (23.5)	741 (20.0)	531 (23.0)	599 (26.1)	303 (25.1)	258 (27.6)	126 (29.7)	
> = 5	5140 (47.3)	2297 (62.1)	735 (31.8)	1161 (50.6)	436 (36.2)	368 (39.4)	143 (33.7)	
**Smoking Status**								
Never	7921 (72.9)	2391 (64.6)	1819 (78.7)	1701 (74.2)	953 (79.0)	736 (78.7)	321 (75.5)	< 0.001
Former Smoker^h^	1889 (17.4)	880 (23.8)	281 (12.2)	379 (16.5)	159 (13.2)	125 (13.4)	65 (15.3)	
Active	259 (2.4)	103 (2.8)	48 (2.1)	61 (2.7)	23 (1.9)	18 (1.9)	6 (1.4)	
Unknown	800 (7.4)	326 (8.8)	162 (7.0)	152 (6.6)	71 (5.9)	56 (6.0)	33 (7.8)	

** For continuous variables, ANOVA followed by Dunnett’s test; for categorical variables, chi-square tests followed by unadjusted pairwise chi-square tests; “White” serves as reference level.

a Race and ethnicity data were collected by self-report in pre-specified fixed categories.

b Other insurance includes military, union, workers compensation etc.

c Assessed based on a diagnosis of alcoholic liver diseases, chronic hepatitis, liver cirrhosis and fibrosis and viral hepatitis (including hepatitis B and C)

d Assessed based on a diagnosis of diabetes mellitus, includes diet controlled and non-insulin dependent diabetes

e Assessed based on a diagnosis of chronic kidney disease in past medical history by ICD-10 coding

f Assessed based on a diagnosis of end-stage kidney disease in past medical history by ICD-10 coding

g Charlson comorbidity index predicts the 10-year mortality for a patient based on age and a number of serious comorbid conditions, such as congestive heart failure or cancer. Scores are summed to provide a total score to predict mortality. The median score of 4 corresponds to a 53% estimated 10-year survival and reflects a significant comorbidity burden for these patients.

^h^ Category includes former smokers who currently are not smoking (n = 1536) plus smokers who did smoke however current smoking status is unknown (n = 353).

### Characteristics of hospitalized patients

The median age for the White patients (72, IQR 61–82) was higher than the median age for all patients (65, IQR 54–77) and for patients in every other race/ethnicity category. The percentage of White patients in the age group 80 and above (32%) was at least twice the percentage of patients in this age group in every other race/ethnicity category (11.1%-16%). White patients had lower rates of Medicaid insurance than all other race/ethnicity groups. There was a low rate of Self-Pay patients for all race/ethnicity groups.

The median score on the CCI for all patients was 4 (IQR 2–7) points, which corresponds to a 53% estimated 10-year survival and reflects a significant comorbidity burden for these patients. There were significant differences by racial/ethnic group in patient’s median score on the CCI (p value < 0.001). CCI score was the highest for White patients with a median score of 6 (IQR 3–8). This corresponds to a 21% estimated 10-year survival.

Hypertension and diabetes were the most common comorbidities for the total sample and for each race/ethnicity. White patients had the highest prevalence of cancer, coronary artery disease, and congestive heart failure, while Black patients had the highest prevalence of hypertension, diabetes and end-stage kidney disease.

### Race/Ethnicity and in-hospital mortality

Unadjusted discharge outcome data by race/ethnicity is presented in **Table 2**. In unadjusted analysis, every racial/ethnic group was significantly less likely to experience in-hospital mortality compared to Whites. After backward elimination, the following were found to be significantly associated with higher in-hospital mortality: increased age group, male sex, Medicare or Self-Pay insurance type, unknown smoking status, and a higher score on the CCI (indicating a greater burden of chronic disease) (**Table 3**).

**Table 2:** Proportion and outcomes of patients by race/ethnicity.

	Total	Non-Hispanic White^a^	Hispanic or Latino	Non-Hispanic Black	Non-Hispanic Multi/Other	Non-Hispanic Asian	Unknown
*Outcome*, *n(%)*	N = 10,869	3700 (34.0%)	2310 (21.3%)	2293 (21.1%)	1206 (11.1%)	935 (8.6%)	425 (3.9%)
**Discharged Alive**	7747 (71.3%)	2539 (68.6%)	1694 (73.3%)	1668 (72.7%)	871 (72.2%)	662 (70.8%)	313 (73.7)
**Death**	2725 (25.1%)	1090 (29.5%)	504 (21.8%)	506 (22.1%)	277 (23%)	248 (26.5)	100 (23.5%)
**Still in Hospital**	6 (0.0006%)	1 (0.0003%)	4 (0.0017%)	0 (0%)	1 (0.0008%)	0 (0%)	0 (0%)
**Transferred**	391 (3.6%)	70.0 (1.9%)	108 (4.7)	119 (5.2%)	57 (4.7%)	25 (2.7%)	12 (2.8%)

a Race and ethnicity data were collected by self-report in pre-specified fixed categories.

**Table 3:** Multivariable Cox proportional hazards model for in-hospital mortality.

Factor	aHR (95% CI)^a^	P value
Age Group, yr		
18–39	ref	ref
40–49	1.11	0.54
50–59	1.29	0.14
60–69	1.55	**0.01**
70–79	1.94	**<0.001**
80 and up	3.15	**<0.001**
Female	0.82	**<0.001**
Race/Ethnicity		
Non-Hispanic White	ref	ref
Hispanic	0.66	**<0.001**
Hospital Day 4	0.85 (0.75–0.98)	**0.028**
Hospital Day 7	0.95 (0.84–1.07)	0.424
Hospital Day 10	1.02 (0.91–1.14)	0.732
Hospital Day 13	1.07 (0.95–1.20)	0.260
Hospital Day 16	1.11 (0.98–1.27)	0.099
Hospital Day 19	1.15 (1.00–1.32)	0.045
Non-Hispanic Black	0.92 (0.82–1.02)	0.106
Multiracial/other	0.61	**0.002**
Hospital Day 4	0.82 [0.69–0.97]	**0.022**
Hospital Day 7	0.92 [0.80–1.06]	0.240
Hospital Day 10	0.99 [0.86–1.13]	0.889
Hospital Day 13	1.05 [0.91–1.21]	0.538
Hospital Day 16	1.09 [0.94–1.27]	0.259
Hospital Day 26	1.20 [1.00–1.46]	0.049
Non-Hispanic Asian	1.11 (0.97–1.28)	0.140
Unknown	1.04 (0.85–1.28)	0.694
Insurance		
Commercial	ref	ref
Medicaid	1.01	0.887
Medicare	1.17	**0.011**
Self-Pay	2.32	**0.004**
Other	1.24	0.310
Smoking Status		
Never	ref	ref
Former	1.04	0.415
Active	1.05	0.778
Unknown	8.36	**<0.001**
CCI Group		
0	ref	ref
1–2	1.04	0.814
3–4	2.02	**0.005**
≥ 5	4.28	**<0.001**

Abbreviation: aHR, adjusted hazard ratio

^a^All variables were used in multivariate analysis

After adjustment for the above covariates, risk of death for Black, Asian, and unknown race/ethnicity patients were not significantly different from White patients. The risk of death for Hispanic and multiracial/other patients was not constant over time, violating the proportional hazards assumption. For the Hispanic group, the risk of death was only significantly different from Whites before hospital day 5 (favored Hispanic) and after hospital day 19 (favored White). Similarly, for the multiracial/other group, the risk of death was only significantly different from Whites before hospital day 5 (favored multiracial/other) and after hospital day 28 (favored White).

## Discussion

This is the largest study to evaluate racial and ethnic differences in in-hospital mortality in diverse patients hospitalized with COVID-19. The size and diversity of the patient population is further supported by the completeness of the record, with 99.9% of patients discharged at the end of follow-up. In this cohort, White patients were older, had more comorbidities, and had higher unadjusted mortality rates than non-White patients. After adjustment for baseline patient characteristics and comorbidities, Black, Asian, Hispanic, and multiracial/other patients had similar adjusted risk of in-hospital mortality compared to White patients.

This study builds on the current literature by examining mortality rates in a population including considerable numbers of patients with Hispanic and non-Hispanic Asian race/ethnicities controlled for appropriate covariates. This study supports the growing body of evidence indicating that population-level disparities in COVID-19 mortality are the result of differential disease incidence and severity of illness rather difference in care for hospitalized patients [22]. For example, although we did not see a disparity in in-hospital mortality for Black patients, they were overrepresented as admitted patients. Blacks made up 12% of the population served but comprised 21.1% of the patients admitted with COVID-19. Additionally, disparities in out-of-hospital deaths related to medical mistrust and health care access may also contribute to inequalities seen at the city and state level. In New York City, out-of-hospital cardiac arrests were 3-fold higher during the 2020 COVID-19 pandemic, and corresponding deaths were more likely to occur in those from non-White race/ethnicities [23].

We found mixed results for Hispanic and multiracial/other patients. This finding is contrasted with age-adjusted, nationwide, publicly reported data showing Hispanics were 2.8 times more likely to experience COVID-19 associated mortality [1]. Additionally, in New York City, the age-adjusted fatality rate per a population of 100,000 is 122 for non-Hispanic White and 238 for Hispanics [24]. Comparison with national and local data is more difficult for those of multiracial and other race/ethnicities. Our findings may point to the utility of expanding access to care for Hispanic patients. Increased access to inpatient care may be a key intervention to reduce disparities in this community.

Our additional findings that increased age, male sex, Medicare or Self-Pay insurance type, unknown smoking status, and a higher comorbidity burden were associated with higher in-hospital mortality are almost all supported by the current literature [7–12]. Unknown smoking status may have been associated with increased mortality as a marker of more severe illness on hospital presentation, and resulting lower likelihood of documentation. Medicaid and Self-Pay insurance were both used as markers of poverty in this study. The lack of association between Medicaid insurance and mortality may be due to the relatively robust Medicaid program in New York.

Our work sits within a greater context of almost two decades of study of health disparities [25]. Overwhelming evidence has shown that, in comparison to Whites, racial and ethnic minorities have worse health outcomes [26–28] that are not explained by genetic differences [29]. Health inequalities are consequences of structural racism and resulting negative social determinants of health, such as educational and employment opportunities, residential segregation, transportation options, food security, and access to care [30–32]. All of these determinants may underlie disparities in COVID-19 mortality. Closing the gap in morbidity and mortality associated with COVID-19 in racial and ethnic minority populations may require a focus on public health, community-based interventions, and structural determinants [33].

### Limitations

This study has several limitations. First, the study population only included patients within the New York City metropolitan area. Second, the data were collected from the electronic health record database. This precluded the level of detail possible with a manual medical record review. Third, the study was limited to one health system; consequently, generalizability to other health care settings may be limited. However, this limitation may be balanced by the size and racial and ethnic diversity of our patient population.
